# Case Report: Early Onset Systemic Lupus Erythematosus Due to Hereditary C1q Deficiency Treated With Fresh Frozen Plasma

**DOI:** 10.3389/fped.2021.756387

**Published:** 2021-12-21

**Authors:** Milica Zecevic, Aleksandra Minic, Srdjan Pasic, Vladimir Perovic, Zoltán Prohászka

**Affiliations:** ^1^Clinical Immunology and Allergy Department, Institute for Health Protection of Mother and Child of Serbia “Dr Vukan Cupic”, Belgrade, Serbia; ^2^Institute of Microbiology and Immunology, Faculty of Medicine, University of Belgrade, Belgrade, Serbia; ^3^Department of Internal Medicine and Haematology, Semmelweis University, Budapest, Hungary; ^4^Research Group for Immunology and Haematology, Semmelweis University—Eötvös Loránd Research Network (Office for Supported Research Groups), Budapest, Hungary

**Keywords:** lupus erythematosus, C1q deficiency, fresh frozen plasma, early-onset autoimmunity, lupus skin involvement

## Abstract

**Background:** Hereditary C1q deficiency is associated with early-onset autoimmunity causing SLE or SLE-like disease as well as increased risk for infections with encapsulated bacteria. It is a rare genetic condition inherited in an autosomal recessive manner, caused by mutations in C1q genes. Treatment and management of this rare disease are very complex and include prophylactic vaccination, antibiotics, and immunosuppressive drugs. There are two possible modalities for the replacement of the missing protein: regular fresh frozen plasma (FFP) administration and allogeneic hematopoietic stem cell transplant because the protein is derived from monocytes. Replacing C1q with FFP is being attempted in some patients with success in controlling the disease and in avoiding flare.

**Case Report:** We report a case of sixteen-month-old girl with ulcerations in her mouth, skin erythema, and elevated liver enzymes. ANAs were positive, antibodies against dsDNA were negative, but she had positive anti-Smith antibodies. Complement complements C3 and C4 levels were normal. Total complement activity, classical pathway (hemolytic test) was deficient and C1q antigen was below the detection limit supporting the presence of C1q deficiency. The girl has pathogenic homozygous nonsense mutation in C1qC gene, Arg69Ter (c205>T). The initial response to corticosteroid therapy was good. Regular fresh frozen plasma infusions keep her disease under control, and we were able to reduce the dose of corticosteroids.

**Conclusion:** Young patients with cutaneous lesions resembling SLE, early onset of autoimmunity, with normal C3, C4, elevated ANAs, and negative anti-dsDNA, C1q deficiency should be suspected and complement screening tests should be done. It is important to exclude secondary C1q deficiency. FFP in our patient seems to be well tolerated, without any side effects, able to control the disease.

## Introduction

C1q is the pattern-recognition molecule in the classical activation pathway of the complement system. The main event in all three complement activation pathways (classical, alternative, and lectin pathway), is the formation of C3 convertase which eventually leads to the activation of terminal pathway, with the formation of membrane attack complex (C5b and complement factors C6-C9). The result of this process is the release of chemotactic factors, opsonization, and lysis of microorganisms (1). C1q has several additional known functions. It facilitates the clearance of apoptotic cells, induces dendritic cell maturation, modulates T cell function, and participates in the negative selection of autoreactive B cells (2). Primary C1q deficiency is a rare genetic condition, inherited in an autosomal recessive manner, caused by mutations in the genes encoding the three different chains of the C1q molecule (C1QA, C1QB, C1QC). To date, there are less than 100 reports of this rare condition in the medical literature. C1q deficiency is associated with early-onset autoimmunity causing SLE or SLE-like disease as well as increased risk for infections with encapsulated bacteria (3). Treatment and management are complex and include prophylactic vaccination, antibiotics, and immunosuppressive drugs (4). There are two possible modalities for the replacement of the missing protein: regular fresh frozen plasma (FFP) administration and allogeneic hematopoietic stem cell transplant because the protein is derived from monocytes. Replacing C1q with FFP is being attempted in some patients with success in controlling the disease and in avoiding flares (5).

## Case Report

A sixteen-month-old girl was referred to Institute for health protection of mother and child of Serbia “Dr Vukan Cupić” due to ulcerations in her mouth, skin erythema, and elevated liver enzymes. The first symptoms appeared six weeks before the admission. She started losing her appetite due to severe mouth ulcerations. She developed malar-like erythema and erythema on her palms. Skin lesions got worse upon sun exposure. Her perinatal anamnesis was normal, she was healthy until she turned 14 months, without any medical history of recurrent or severe infections. She was regularly vaccinated for her age according to national protocols (bacillus Calmette-Guérin vaccine, hepatitis B vaccine, DTP vaccine, MMR vaccine) but was not given the conjugated anti-pneumococcal vaccine. On admission to our hospital, she was febrile (38°C), tachycardic and eupneic. She had malar rash, erythema on her philtrum, and periauricular erythema with severe ulcerations in her mouth. She had vasculitis-like lesions on her palms. She had no clinical signs of arthralgia or arthritis and the rest of the physical examination was normal. Laboratory test showed: high erythrocytes sedimentation rate, normocytic Coombs negative anemia (Hgb 95 g/l, MCV 80 fL), leukopenia with lymphopenia (leucocyte count 3,85 × 10^9^/l, lymphocyte count 770 × 10^9^/l), and elevated liver enzymes with normal liver function tests. C- reactive protein and procalcitonin were in the reference range. Coagulation screening tests were also normal, showing no elevation of d-dimers. Renal function tests were normal. Hemoculture and urine culture were sterile. Infective etiology of hepatitis was excluded. Radiological examinations were all normal. She was given broad-spectrum antimicrobial therapy. Further immunological investigations were performed due to suspected early onset of autoimmunity. Immunoglobulin levels were in the reference range for her age. ANAs were positive (ANA-HEp2 IgG human cells IIF) at 1:640 titer (nucleoplasm with a speckled pattern). Antibodies against dsDNA were negative, but she had positive anti-Smith antibodies >300 U/ml (reference range <18 U/ml, IgG, ELISA). Complement C3 component was 1,8 g/l (reference range 0,9–1,8 g/l) and C4 component was 0,4 g/l (reference range 0,15–0,55 g/l). Total complement activity, classical pathway (hemolytic test) was deficient, 7 CH50/ml (reference range 48–103 CH50/ml), and C1q antigen was below the detection limit (0 mg/l), supporting the presence of C1q deficiency (reference range 60–180 mg/l). Anti-C1q IgG autoantibody was negative (1 U/ml, reference range <52 U/ml). Alternative and lectin pathway activities were in the reference range or elevated with elevated sC5b9 marker level also indicating fully operating alternative and lectin pathways, with activation of the terminal pathway (Table 1). Pathohistological examination of the skin biopsy showed interface dermatitis with IgM deposits in the zone of the basal membrane, characteristic of cutaneous lupus erythematosus. The girl was diagnosed with systemic lupus erythematosus having score 17 of EULAR/ACR 2019 diagnostic criteria (positive ANA with leukopenia, oral ulcers, acute cutaneous lupus and positive anti-Smith antibody). According to American College of Rheumatology Criteria (ACR 1997) she had 6 of 11 criteria at the time of diagnosis (malar erythema, oral ulcers, photosensitivity, leucopenia and lymphopenia, positive ANA, and anti-Smith antibody). She was given methylprednisolone (2 mg/kg) intravenously. The initial response to corticosteroid therapy was good: patient became afebrile after a few days, oral ulcers started to heal and erythema on her face started to disappear. Laboratory tests showed significant improvement (normal erythrocyte sedimentation, normalization of anemia, leucopenia and serum transaminases levels). The attempt to reduce the dose of corticosteroids led to the cutaneous flare of the disease.

**Table 1:** Complement testing results.

Total complement activity, classical pathway (hemolytic test)	7 CH 50/ml (reference range 48–103 CH50/ml)
Total complement activity, alternative pathway (WIELISA-Alt)	75% (reference range 70–125%)
Total complement activity, lectin pathway (WIELISA-LP)	227% (reference range 25%−125%)
Complement C3	1,96 g/l (reference range 0,9)
Complement C4	0,4 g/l (reference range 0,15–0,55 g/l)
Factor H antigen	739 mg/l (reference range 250–880 mg/l)
Complement factor I antigen	143 % (reference range 70–130%)
Complement factor B antigen	127 % (reference range 70–130%)
C1q antigen	0 mg/l (ref. 60–180 mg/l)
Anti C1q IgG autoantibody	1 U/ml (ref. <52)
sC5b-9 (terminal component complex)	645 ng/ml (reference range 110–252 ng/ml)

**Figure 1:**
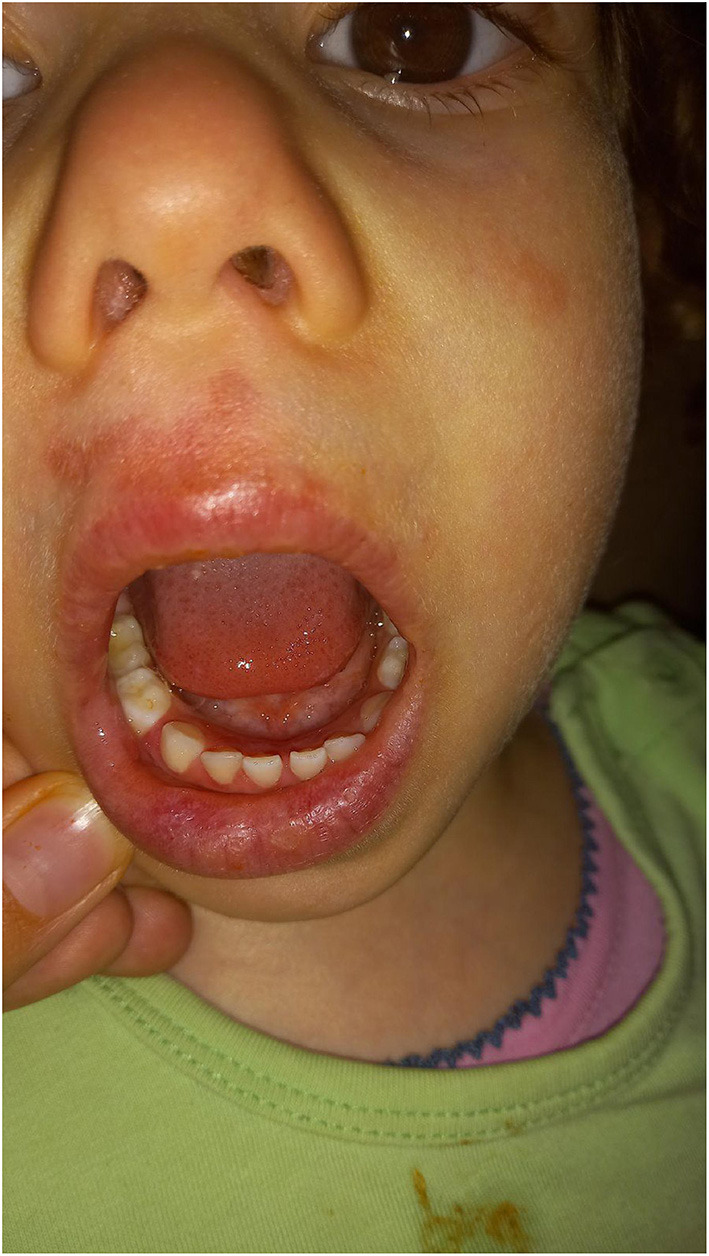
Redness around girl's philtrum and aphthous changes on her lips.

**Figure 2:**
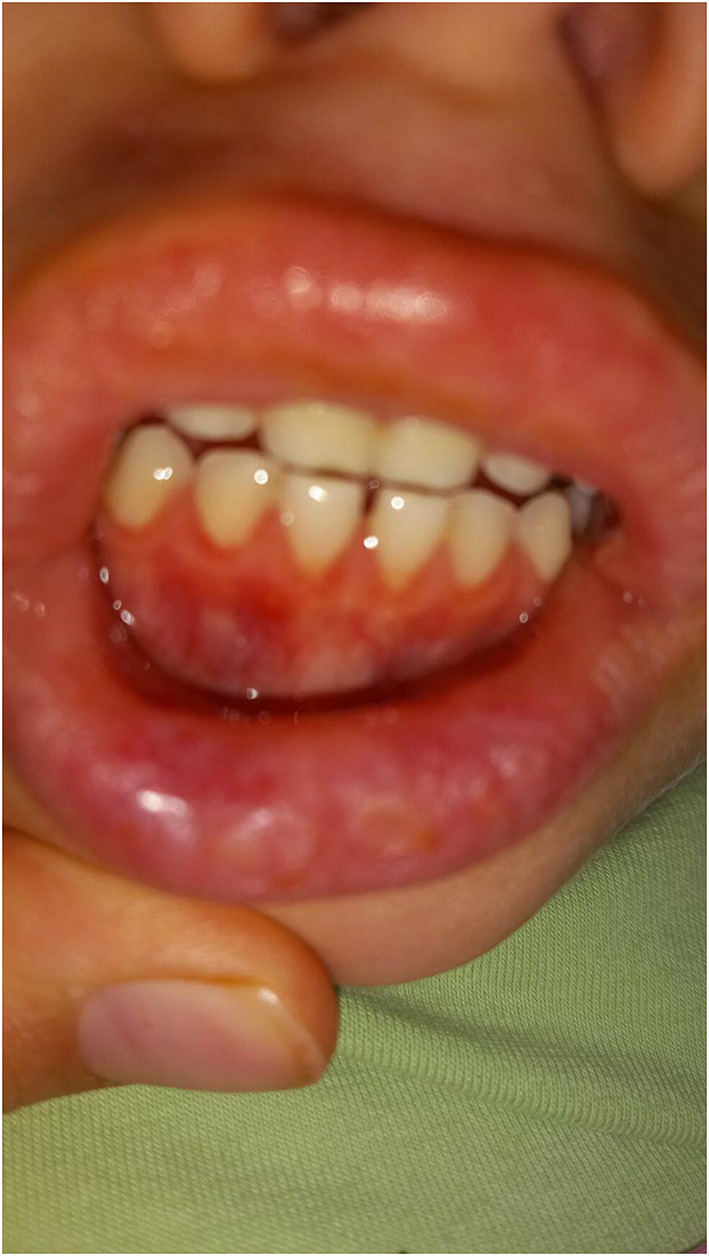
Aphthous changes on the inner side of girl's mouth and on her lips.

**Figure 3:**
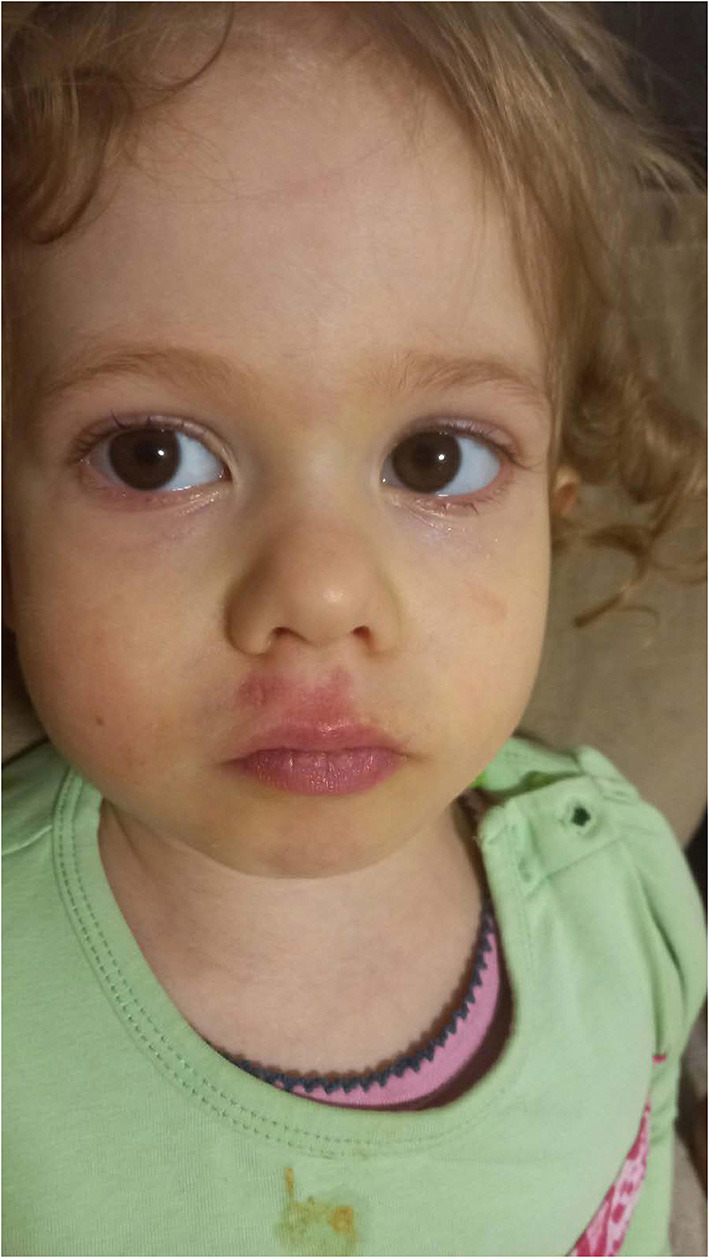
Pale face, with philtrum erythema.

Replacement of the missing protein was attempted with regular FFP treatment. The initial dose of FFP was 15 ml/kg, and it was well tolerated. Cycles of treatment were administered every three weeks for five consecutive days. She developed urticaria after the second FFP infusion which disappeared after treatment with antihistamine drug. Patient has been treated with FFP for over a year now, and the dose of corticosteroids was tapered to 0,3 mg/kg every day. She is in good general health now, slowly growing, with only little flares of oral ulcers and skin erythema just on her philtrum a few days before the next cycle of FFP (Figures 1–3). She did not develop any other organ involvement during FFP treatment and she has regular examinations every three weeks. Each time she comes to the hospital we perform a detailed skin and mucous membrane examination, nephrological tests (urine analysis for proteinuria and calciuria). A big part of her follow up is her growth rate which is improving as we are lowering the corticosteroid dose. ANA and anti-Smith antibodies can not be used for the disease activity follow up.

Allogeneic hematopoietic stem cell transplantation was considered, but is postponed for now because she does not have any other manifestation of the disease and it can be well-controlled with FFP. Future goal is to lower the dose of corticosteroids. Whole exome sequencing was performed, and it showed that the girl has pathogenic homozygous nonsense mutation in C1qC gene, p.Arg69Ter (c205 > T).

## Discussion

Clinical presentation of C1q deficiency ranges from severe or mild recurrent bacterial infections to SLE-like disease (3 ACR criteria) or SLE with severe neurological and kidney involvement. Some individuals are without any symptoms of the disease (3). Other early complement component deficiencies (C1r, C1s, C4, C2) can be the cause of SLE but C1q is the most predisposing factor. Walport et al. found that 93% (38 out of 41 patients) with C1q deficiency have clinical features closely related to SLE (6). In the more recent survey by Stegart et al. about 77.5% (total number of 71 patients) of C1q deficient patients have SLE or SLE-like disease according to ACR criteria which are most likely due to the stricter use of classification criteria (3). The median age of disease onset is around 5 years, which makes these patients much younger compared to sporadic SLE. Our patient was only fourteen months old at the disease onset. Men and women are equally affected since the disease is inherited in an autosomal recessive manner. C1q deficient patients significantly more often have severe skin involvement (malar rash/discoid rash) and oral ulcers compared to sporadic SLE (3). Binding to apoptotic debris and accelerating the removal of immunocomplexes, C1q protects the organism from overexposure to autoantigens and from the development of autoimmunity. Lack of C1q thus leads to deficient clearance of apoptotic cells which acts as a source of autoantigens; defective negative selection of autoreactive B cells and distorted cytokine production (4, 7). Cutaneous manifestations are probably caused by over-reactive apoptosis of keratinocytes upon sun exposure and defective clearance of the debris (8). Our patient had a face rash resembling malar rash and extensive oral ulcers enabling her to eat or drink. Oral ulcers in sporadic SLE are not painful and usually are only found on the hard palate. Skin manifestations also included small erythematous lesions on her palms resembling skin vasculitis. At the time of diagnosis, we did not find any neurological or kidney involvement. Renal and neurological manifestations seem to occur with the same prevalence in C1q deficiency and sporadic SLE (3). Regarding the immunological features of this rare disease, it is important to notice that these patients have normal C3 and C4 levels. Total complement activity, classical pathway (hemolytic test) was deficient, and C1q antigen was below the detection limit which led us to the diagnosis. C1q deficient patients have anti-Smith antibodies significantly more often than patients with sporadic SLE and they significantly less often have positive anti-dsDNA (3). Corticosteroids are the most frequently reported drugs used in the treatment. They are highly effective but severe flare-ups appear upon dose reduction or withdrawal even if other immunosuppressive agents (hydroxychloroquine, methotrexate, azathioprine) are used (9, 10). Our patient showed significant clinical improvement and normalization of laboratory tests during high dose corticosteroid treatment. Besides the immunosuppressive drugs, the other approach in the treatment includes the replacement of the missing protein with FFP at regular intervals. There are only a few reports about the use of FFP in C1q deficiency (5, 11, 12). FFP treatment can last for years so it is important to take the side effects into account. The risk of transfusion-related infections is not high regarding the standard use of donor screening tests. Severe allergic reaction after FFP infusion is rarely reported and urticarial reactions are easily controlled with antihistamine drugs. Acute hemolytic reactions, hypocalcemia, and transfusion-related acute lung injury are extremely rare side effects of FFP infusions (13). One of the most important limitations of this treatment could be the development of anti-C1q antibodies. There are no guidelines about whether the patients should be screened for C1q antibodies development during FFP treatment or a clinician should only monitor the efficacy of the FFP treatment. C1q is synthesized by bone marrow-derived monocytes. Studies in mice suggested that C1q deficiency might be treated with HSCT (14). The first allogeneic hematopoietic stem cell transplantation was successfully performed, in a 16-year-old boy with C1q deficiency from a healthy matched sibling donor (15). To date, there are only a few reports about the attempts in HSCT in these patients with variable outcomes (16). HSCT does restore C1q production but there is a considerable risk of transplant-related complications and mortality. In successful cases, it is shown that HSCT can be a definitive cure. Whole-exome sequencing showed previously described pathogenic mutation in the C1QC gene according to ClinVar. The girl has homozygosity for C1qC p.Arg69Ter (c205 > T) mutation which is a pathogenic nonsense mutation. To our knowledge, this is the third patient from Kosovo and former Yugoslavia to have such a mutation (17). The parents of the girl are not consanguineous to their knowledge, but they both come from Kosovo, and they cannot exclude the ancient common ancestor. All three patients with this mutation have the same clinical and immunological characteristics (17, 18). In conclusion, young patients with cutaneous lesions resembling SLE, early onset of autoimmunity, with normal C3, C4, elevated ANAs, and negative anti-dsDNA, C1q deficiency should be suspected and complement screening tests should be done. It is also important to analyze anti-C1q antibody to exclude secondary C1q deficiency. FFP in our patient seems to be well tolerated, without any side effects, able to control the disease.

## Data Availability Statement

The original contributions presented in the study are included in the article/supplementary material, further inquiries can be directed to the corresponding author/s.

## Ethics Statement

Written informed consent was obtained from the individual(s), and minor(s)' legal guardian/next of kin, for the publication of any potentially identifiable images or data included in this article.

## Author Contributions

All the authors have contributed in the diagnosis and treatment as well as writing of this case report.

## Conflict of Interest

The authors declare that the research was conducted in the absence of any commercial or financial relationships that could be construed as a potential conflict of interest.

## Publisher's Note

All claims expressed in this article are solely those of the authors and do not necessarily represent those of their affiliated organizations, or those of the publisher, the editors and the reviewers. Any product that may be evaluated in this article, or claim that may be made by its manufacturer, is not guaranteed or endorsed by the publisher.

## References

[1] Walport MJ. Complement. First of two parts. N Eng J Med. (2001) 344:1058–66. 10.1056/NEJM200104053441406 11287977

[2] Nayak A Ferluga J Tsolaki AG Kishore U. The non-classical functions of the classical complement pathway recognition subcomponent C1q. Immunol Lett. (2010) 131:139–50. 10.1016/j.imlet.2010.03.012 20381531

[3] Stegart M Bock M Trandelburg M. Clinical presentation of human C1q deficiency: how much of a lupus. Mol Immunol. (2015) 67:3–11. 10.1016/j.molimm.2015.03.007 25846716

[4] Botto M Walport MJ. C1q autoimmunity and apoptosis. Immunobiology. (2002) 205:395–406. 10.1078/0171-2985-00141 12396002

[5] Ekinci Z Ozturk K. Systemic lupus erythematosus with C1q deficiency: treatment with fresh frozen plasma. Lupus. (2018) 27:134–8. 10.1177/0961203317741565 29113537

[6] Walport MJ Davies KA Botto M. C1q and systemic lupus erythematosus. Immunobiology. (1998) 199:265–85. 10.1016/S0171-2985(98)80032-6 9777411

[7] Korb LC Ahearn JM. C1q binds directly and specifically to surface blebs of apoptotic human keratinocytes: complement deficiency and systemic lupus erythematosus revisited. J Immunol. (1997) 158:4525–8. 9144462

[8] Macedo AC Isaac L. Systemic lupus erythematosus and deficiency of early components of the complement classical pathway. Front Immunol. (2016) 7:55. 10.3389/fimmu.2016.00055 26941740 PMC4764694

[9] Kallel-Sellami MA Baili-Klila LI Zerzeri Y Laadhar L Blouin J Abdelmoula MS. Pediatric systemic lupus erythematosus with C1q deficiency. Ann N Y Accad Sci. (2007) 1108:193–6. 10.1196/annals.1422.021 17893985

[10] Higuchi Y Shimizu J Hatanaka M Kitano E Kitamura H Takada H. The identification of a novel splicing mutation in C1qB in Japanese family with C1q deficiency: a case report. Pediatr Rheumatol. (2013) 11:41. 10.1186/1546-0096-11-41 24160257 PMC3874733

[11] Topaloglu R Taskiran EZ Tan C Erman B Ozaltin F Sanal O. C1q deficiency: identification of a novel missence mutation and treatment with fresh frozen plasma. Clini Rheumatol. (2012) 31:1123–6. 10.1007/s10067-012-1978-4 22576477

[12] Mehta P Norsworthy PJ Hall AE Kelly SJ Walport MJ Botto M. SLE with C1q deficiency treated with fresh frozen plasma: a 10-year experience. Rheumatology (Oxford). (2010) 49:823–4. 10.1093/rheumatology/kep387 19965977

[13] Pandey S Vyas GN. Adverse effects of plasma transfusion. Transfusion. (2012) 52:65S−79S. 10.1111/j.1537-2995.2012.03663.x 22578374 PMC3356109

[14] Cortes-Hernandez J Fossati-Jimack L Petry F Loos M Izui S Walport MJ. Restoration of C1q levels by bone marrow transplantation attenuates autoimmune disease associated with C1q deficiency in mice. Eur J Immunol. (2004) 34:3713–22. 10.1002/eji.200425616 15517607

[15] Arkwright PD Riley P Hughes SM Alachkar H Wynn RF. Successful cure of C1q deficiency in human subjects treated with hematopoietic stem cell transplantation. J Allergy Clin Immunol. (2014) 133:265–7. 10.1016/j.jaci.2013.07.035 24035158

[16] Olsson RF Hagelberg S Schiller B Ringdén O Truedsson L Åhlin A. Allogeneic hematopoietic stem cell transplantation in the treatment of human C1q deficiency: the Karolinska experience. Transplantation. (2016) 100:1356–62. 10.1097/TP.0000000000000975 26516671

[17] Schejbel L Skattum L Hagelberg S Åhlin A Schiller B Berg S. Molecular basis of hereditary C1q deficiency-revisited: identification of several novel disease-causing mutations. Genes Immun. (2011) 12:626–34. 10.1038/gene.2011.39 21654842

[18] Slingsby JH Norsworthy P Pearce G Vaishnaw AK Issler H Morley BJ. Homozygous hereditary C1q deficiency and systemic lupus erythematosus. A new family and the molecular basis of C1q deficiency in three families. Arthritis Rheum. (1996) 39:663–70. 10.1002/art.1780390419 8630118

